# Eligibility, the ICF and the UN Convention: Australian perspectives

**DOI:** 10.1186/1471-2458-11-S4-S6

**Published:** 2011-05-31

**Authors:** Ros Madden, Nick Glozier, Elias Mpofu, Gwynnyth Llewellyn

**Affiliations:** 1 Australian ICF Disability and Rehabilitation Research Program, Faculty of Health Sciences, The University of Sydney, Sydney, Australia; 2 Disciplines of Psychological Medicine and Sleep, Sydney Medical School, The University of Sydney, Sydney, Australia; 3 Faculty of Health Sciences, The University of Sydney, Sydney, Australia

## Abstract

The UN Convention on the Rights of Persons with Disabilities, in Australia, acts as a philosophical and moral statement and framework guiding integrated and strategic policy across the nation. Broad policy agreement has been reached by governments, and both the government and non-government sectors are developing strategies for implementation or evaluation. There is however a need for a more integrated approach to disability policy and information, reflecting all three components of the Italian project:

• legislation and a high level philosophical framework and policy guide;

• a technical framework that can underpin specific policies and programs aiming to achieve the major goals; and ,

• a language and set of tools, relating to both the above, that provide infrastructure for assessment methods and information systems.

The International Classification of Functioning, Disability and Health (ICF) is the ideal tool to support the latter two components, consistent with the UN Convention. While the ICF has been used as the basis for national data standards, in population surveys and in the national data collection on disability support services, there is considerable scope for greater use of it, including using all domains of the Activities and Participation and the Environmental Factors component for policy, information and service provision, to advance a disability-inclusive society. Information available from the income support system and from generic services could be enhanced by reference to the ICF components. It would be of significant national value in Australia, especially as a ‘continuum of care’ is desired, if consistency of concepts and information were expanded across health and social welfare sectors. It would then be possible to obtain consistent data from health, aged care, disability and community services systems about key aspects of health and functioning, building a consolidated picture of access and experience across these sectors. Without attention to all three components of the Italian project and continuing effort to meet the challenges identified in this paper, it will not be possible to determine whether the goals of Australia’s National Disability Agreement or the ambitions of the Convention are achieved.

## Background

This paper discusses eligibility for and information from two major disability-related national programs in Australia, with reference to the International Classification of Functioning, Disability and Health (ICF) and the UN Convention on the Rights of Persons with Disabilities. According to the ICF, ‘Functioning … is an interaction or complex relationship between the health condition and …environmental and personal factors’ [1]. Components of functioning and disability in the ICF are: Body Function and Structure, and Activities and Participation. Disability, thus, ‘is the umbrella term for any or all of: an impairment of body structure or function, a limitation in activities, or a restriction in participation’ relating to health conditions and environmental factors [2]. An estimated 20% of the 22 million-strong Australian population had a disability in 2003 (using this broad multidimensional concept); 6.3% of the population had a ‘severe or profound core activity limitation’, meaning that they needed assistance with self care, mobility and/or communication [3,4].

The paper first outlines some key components of the Australian disability system, focussing on two major national programs – for income support and specialist disability support services. Eligibility and assessment for these programs are described and discussed. Information available from the programs is presented, aspects of information design are discussed, and potential improvements to information – collection, recording and analysis – are suggested. A concluding discussion relates the three components of the Italian project [5] to the Australian experience.

The design of disability programs, services or benefit schemes is a major undertaking and this paper does not discuss other major design features which may also affect eligibility – such as means testing, benefit levels, or funding methods. Nor, in discussing the Australian system, can the paper cover all closely related programs – such as anti-discrimination measures, aged care and rehabilitation – which require integration with the programs discussed here. Australian workers compensation schemes and transport accident insurance schemes are state-based contributory schemes and often have a ‘fault’ component; these schemes are not discussed in this paper which focuses on national programs defined and funded by governments.

## Overview of the Australian disability system

Broadly, formal services and assistance for people with disability in Australia comprise:

• income support;

• specialist disability support services; and

• relevant generic services, such as health, education and housing (some of which have disability sub-programs).

It must be remembered, in the following discussion of the first two categories of services, that most assistance received by people with a disability is provided by family and friends (e.g. [6,7]).

As a federation, Australia has a national government and eight state and territory governments. In 2008, the Council of Australian Governments agreed to a new National Disability Agreement to improve and expand services for people with disability, their families and carers [8]. In the Agreement, all Australian governments committed to the ‘overarching aspiration’ (Clause 6) that:

‘All aspects of the National Disability Agreement contribute to, or measure progress towards:

“People with disability and their carers have an enhanced quality of life and participate as valued members of society” ’

The Agreement sets out the roles and responsibilities of governments and commits them to contributing to ‘economic participation and social inclusion’ and to ‘people with disability and enjoying choice, wellbeing and the opportunity to live as independently as possible’; families and carers are to be ‘well supported’.

The Australian Government is developing a National Disability Strategy, in consultation with the community, disability and carer peak bodies, employers, industry experts and state and territory governments. This is seen as an important mechanism to ensure that the principles underpinning the United Nations Convention on the Rights of Persons with Disabilities, ratified by the government, are integrated into policies and programs affecting people with disability, their families and carers (e.g. [9]).

## Income support for people with a disability

Under the National Disability Agreement, income support, targeted to the needs of people with disability, their families and carers is a responsibility of the national (Commonwealth) government. The national income support schemes are financed by taxes.

### The Disability Support Pension: overview of recipients

The main income support program for people of working age and relevant to people with a disability is the Disability Support Pension (DSP), for which there was an appropriation of $9.37 billion in 2007-08 [10]. (There are also payments and schemes such as carer allowances and payments and a mobility allowance, not discussed here.)

In June 2008, the DSP population was 732,367 [11]; 56.5% were male and 43.5% female. The proportion of male recipients has steadily diminished since June 2003; average duration on a DSP is 11.3 years. As in all Organisation for Economic Cooperation and Development (OECD) countries, the proportion and absolute number of the population receiving disability-related income support has been increasing over recent decades – from approximately 140,000 in 1972 to over 730,000 in 2007-08 [12]. There is a suggestion that, as with some other countries, this had reached a plateau in the mid 2000s following significant policy changes to limit the growth in disability income support over this latter period. In particular, the recent steadying in numbers is considered related to the changes in policy and assessment methods [12-15]. Policy factors associated with these trends are multi-faceted and include: changes to eligibility criteria and assessment methods; changes to the means test (for instance, treatment of partner’s income); changes to related schemes (for instance, the phasing out of widows’ benefits, and changes to unemployment benefits and the qualifying age for women for the age pension); and recent efforts to reduce disincentives to work.

### Eligibility for the DSP: two stages of assessment

The DSP is ‘intended to ensure that people with disabilities have adequate levels of income and maximum opportunities to participate in society’ [11].

Under Section 94 of the Social Security Act 1991 [16] a person is eligible for the DSP if

• the person has a ‘physical, intellectual or psychiatric impairment’,

• the person's impairment is of 20 points or more under the Impairment Tables attached to the Act, in Schedule 1B,

• the person has a ‘continuing inability to work’ (not able to work for 15 hours+ per week, at or above the relevant minimum wage, or be reskilled for such work for 2 years+), or is working under the Supported Wage System, and

• is aged 16+ and under Age Pension age, and satisfies certain residential requirements.

A means test applies (Section 1064). People who are permanently blind qualify automatically (under Section 95) and are not subject to the means test. In deciding whether or not a person has a ‘continuing inability to work’, specific environmental factors are *not* to be taken into account, including the availability to the person of training or work in the person’s local labour market.

The award of DSP is essentially a two step process. Firstly, there must be an ‘impairment’; this is usually identified initially as a medically defined condition which represents the primary ‘medical category’ that is recorded on a recipient's record at time of the grant or review of a DSP. The four most common categories are: Musculo-skeletal and connective tissue conditions; Psychological/Psychiatric conditions; Intellectual/Learning difficulties; and Circulatory systems [11]. This is followed by two assessments which have now been combined into one process [17,18]:

• the assessment of ‘impairment’, and

• the assessment of ‘continuing inability to work’ or (its administrative name) ‘job capacity’.

This assessment is conducted by one of 1,700 Job Capacity Assessors – trained and approved allied health professionals (occupational therapists, social workers etc). They interview the client, review the medical and other evidence and complete a Job Capacity Assessment (JCA) Report [17].

### Assessment of ‘work-related impairment’

Tables for the assessment of ‘work-related impairment’ for the DSP are set out in Schedule 1B of the Social Security Act. These are designed to assess whether the person being considered for DSP ‘meets an empirically agreed threshold in relation to the effect of their impairments, if any, on their ability to work’. The introduction to the Tables states that they ‘represent an empirically agreed set of criteria for assessing the severity of functional limitations for work-related tasks and do not take into account the broader impact of a functional impairment in a societal sense’.

The 22 Tables could be described as body-system based. The assessor considers each medical condition and its related ‘functional impairments’ for any body system affected, and determines an impairment rating. Any diagnosed medical condition considered must be treated and stabilised before assessment, and be considered ‘permanent’ i.e. unlikely significant functional improvement within the next two years. In scoring the related level of impairment, the assessor first scores the ‘loss of function’ for each body system, then adds separate scores (across systems and conditions) to give a single score for ‘work-related impairment’. Double counting is to be avoided, for instance where three conditions contribute to one ‘loss of function’; defining the core loss of function can be difficult: for instance an impaired lung function (measured using Forced Expiratory Volume) can reduce the exercise tolerance.

### Assessment of job capacity

The next stage of assessment if a person is applying for the DSP or undergoing a medical review of DSP is the JCA, which provides ‘comprehensive work capacity assessment, combining referral to employment and related support services with assessment of work capacity for income support purposes’. The vast majority (83%) of applicants are now referred to some form of employment service [19]. It is said that the assessment of ‘ability to work’ focuses on competencies and assists people to use support services which help them find and maintain employment. As well as a range of judgments by the trained assessors, use is made of a ‘Job Seeker Classification Instrument’ whose factors include a range of personal, social and environmental factors and a ‘disability/medical conditions’ factor [20].

The JCA Program was introduced in Australia in July 2006 and has been favourably observed by the OECD: ‘the new comprehensive JCA is a promising step as an integrated assessment aimed at earlier intervention, and the last step in a shift from a medical to a functional view of disability. The dual assessment and referral role could develop into its key strength’ [15].

Nevertheless, a review was undertaken by the new national government in 2007. While resulting in general endorsement, a number of improvements were recommended. Issues raised by stakeholders during the review include: ‘the inflexibility and complexity of current program and policy settings, in particular claims that there is too much focus on rules, barriers, program boundaries and contract requirements’ [19]. In 2007-08 the Administrative Appeals Tribunal received 542 applications for review of decisions relating to the DSP, almost 25% of all applications relating to social security [21].

### The relationship of ‘impairment’ in Schedule 1B to the ICF

The broad relationship between the Impairment Tables of Schedule 1B and the ICF is explored in Table 1, which compares the table headings, the content of the impairment ratings of Schedule 1B, and the ICF dimensions of Body Function and Structure, and Activities and Participation.

**Table 1:** The relationship of Schedule 1B of the Social Security Act with ICF (examples)

**Schedule 1B of SS Act** (Table headings for assessment of ‘work related impairment’ for DSP)	**ICF Body Functions & Body Structures** (Chapter headings)	**ICF Activities and Participation** (Chapters mentioned in Schedule 1B assessment instructions)
1. Loss of cardiovascular and/or respiratory function: exercise tolerance	4 (BF) Functions of the cardiovascular, immunological and respiratory systems 4 (BS) Structures of the cardiovascular, immunological and respiratory systems	4. Mobility 6. Domestic life 8. Major life areas (*manualwork*) 9. Community, social and civic life (*recreation*)

3. Upper limb function	7 (BF) Neuromusculoskelatal and movement-related functions^1^ 7 (BS) Structures related to movement	–

5. Spinal function (*assessed mainly on movement*)	7 (BF) Neuromusculoskelatal and movement-related functions 1 (BS) Structures of the nervous system (for spinal cord) 7 (BS) Structures related to movement (for vertebral column)	4. Mobility

6. Psychiatric impairment 7. Alcohol and drug dependence	*Both the AMA Guides and the SS Act Schedule describe the assessment in terms of ‘health conditions’ (assessed by psychiatrists with reference to DSM-IV) and to various domains of Activities and Participation in ICF i.e. not ‘impairment’*	2. General tasks and demands 3. Communication 6. Domestic life 7. Interpersonal interactions and relationships 8. Major life areas 9 Community, social and civic life

8. Neurological function: memory, problem solving, decision making abilities & comprehension 9. Communication function- receptive and expressive language competency^1^ 10. Intellectual disability	1 (BF) Mental functions 1 (BS) Structures of the nervous system	1.Learning and applying knowledge 2. General tasks and demands 3. Communication 5. Self care 8. Major life areas (e.g. financial transactions)

13. Visual acuity in the better eye 14. Miscellaneous eye conditions 15. Visual fields	2 (BF) Sensory functions and pain^2^ 2 (BS) The eye, ear and related structures	–

Notes:

1. ICF Chapter 7(BF) does not distinguish upper and lower limbs, while 7(BS) does so.

2. The ICF chapters have blocks relating separately, e.g. Chapter 2 (BF) Sensory functions and pain has separate blocks on ‘seeing and related functions’, ‘hearing and vestibular functions’ and ‘additional sensory functions’.

While the Social Security Act Schedule 1B states that ‘impairment’ is being assessed, the assessment in fact often involves, in ICF terms, consideration of a range of activity limitations and participation restrictions. Phrases such as ‘difficulties with everyday activities’ are used frequently in Schedule 1B in the instructions on how to assess the severity of impairment. The exceptions are respiratory function, visual, hearing and upper extremity impairments, where there appear to be accepted medical scales for gauging severity of impairments.

The use of the term ‘impairment’ in Schedule 1B is thus quite different from the term as now understood in the international standard, the ICF. First, the term is used in varied ways: ‘impairment type’; ‘work-related impairment’; ‘functional impairment’. Second, in the Impairment Tables, assessment involves not only health condition, impairment of Body Function and Structures, but often also Activity limitations or Participation restrictions.

Does the mixed concept matter? In the Tables, for instance, is it just that ‘severity’ measures do not exist for most body functions and structures and there is no alternative to creating a mixed concept by also assessing activities and participation?

There seem to be two main reasons why the mixed concepts do indeed matter and why clarification of concepts (at least) would be an advantage:

• lack of clarity can be accompanied by a lack of ability to extract meaningful data from the records, hence leading to a lack of understanding of anything more than eligibility – there is no real health profile of recipients and hence a related loss in understanding of trends; and

• the more the Impairment Tables look at broad aspects of functioning, the more they are potentially duplicating the JCA.

The situation with the JCA is also confusing. Although specific environmental factors are *not* to be taken into account, in determining someone’s ability to work in practice, the main focus of the JCA is in identifying any barriers the person may have to finding and keeping a job. Thus, both personal and potentially environmental factors play a role in the assessment.

### Mental health and disability

A very common condition associated with DSP recipience in most OECD countries is psychiatric disorder, which impairs exactly those functions most required in modern workplaces – concentration, cognitive function, energy, motivation, and social interaction [22]. However, the complexity of relationships among mental health conditions and work impairment causes difficulty in assessment. Firstly, the level of work-related impairment sufficient to be eligible for DSP in the Schedule 1B Impairment Tables is defined by ‘significant interference with interpersonal or workplace relationships with serious disruption of work attendance or ability to work’ or, in the case of substance dependence ‘Dependence… which is sufficient to cause prolonged absences from work’, thus introducing circularity. Secondly, relying upon diagnosis helps little in this area as there is no direct relationship between the presence of psychiatric disorder and employment status or work performance. In the UK, for example, the employment rates of women with psychiatric disorder range from 42% (phobias) to 59% (panic disorder), compared to employment rates of 62% overall [23]. There is extensive evidence that contextual and personal factors are primary determinants of DSP recipience in these conditions regardless of diagnosis and impairment [22].

This would suggest that moving towards an assessment using the ICF framework may have advantages in mental health and disability. For instance, the extra effort that those with psychiatric disorder report having to make, in order to do their work, e.g. to overcome poor motivation or anxious cognitions, can be assessed [24], as could impairments in concentration and cognitive function, through simple neuropsychological tests. This approach may help disentangle the underlying impairment from the commonly cited causes of difficulty at work such as perceived pressure or relationship issues, and could set up a policy-relevant research agenda in this area.

## Specialist disability support services

Under the National Disability Agreement, governments in Australia share responsibility for specialist disability support services. The national government funds employment services and state and territory governments fund and provide other services such as support in the home and community.

### Eligibility

The National Disability Agreement is silent on eligibility for specialist disability support services [8]. This is in contrast to the previous national agreement (the Commonwealth State/Territory Disability Agreement 2003-07) which stated that, for its purposes, ‘people with disabilities’ means people with disabilities attributable to an intellectual, psychiatric, sensory, physical or neurological impairment or acquired brain injury (or some combination of these) which is likely to be permanent and results in substantially reduced capacity in at least one of the following:

• self care/management

• mobility

• communication

requiring significant ongoing and/or long-term episodic support and which manifests itself before the age of 65.

Neither the new nor the older agreement specifies national eligibility assessment processes. However, under the new Agreement, all Australian governments have agreed to concentrate efforts to achieve reforms in ten priority areas including ‘Improved Access to Disability Care – Systems that improve access to disability care and ensure people are referred to the most appropriate disability services and supports, including consideration of single access points and national consistent assessment processes in line with nationally agreed principles’.

### Support services: data design and the ICF

National data about Australia’s disability support services and recipients are collated and analysed each year in a National Minimum Data Set comprising 14 questions about service outlets (for instance location, size and service type) and 17 questions about service users. One question (and data item) relates to people’s ‘support needs’, based on the ICF Activities and Participation domains and also on the Australian disability population survey question on frequency of need for support.

**Figure 1:**
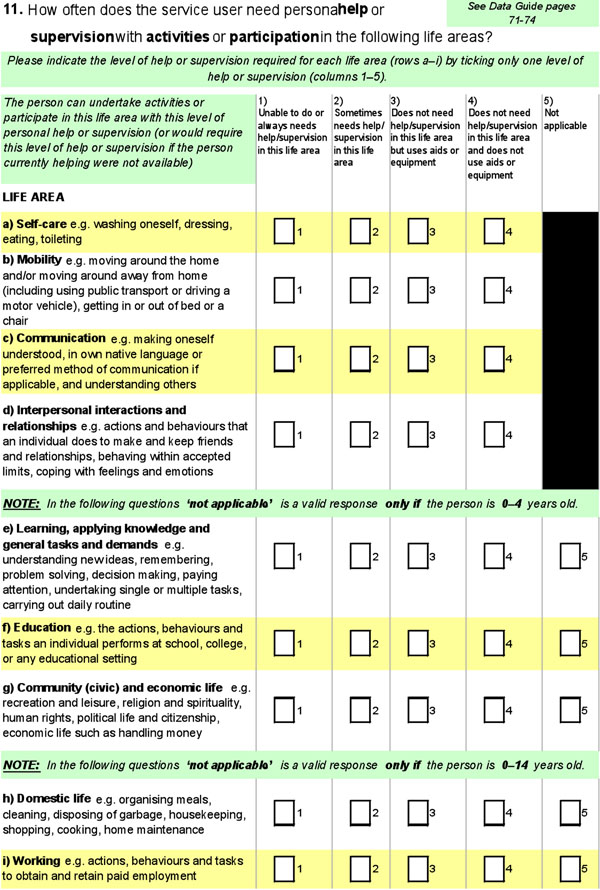
Question on frequency of need for support

In developing the question about ‘support needs’ (the need for personal help or supervision), the design parameters were that the data item should be comparable with the main population data, enable the results from the main assessment tools in the field to be recorded using it, and be consistent with national data standards based on the ICF [25,26]. The question resulting from development, consultation and testing was a ‘data capture matrix’ comprising rows reflecting the ICF Activities and Participation domains, and columns reflecting the national survey question (see Figure 1). For each of 9 life domains based on the ICF Activities and Participation domains, there are (essentially) three simple categories for the frequency of need for support: needs no help/supervision – with or without aids; sometimes needs help/supervision; always needs help/supervision. This simple two-dimensional data capture framework appears to have useful and desirable statistical qualities, and enables the collection of data from thousands of services using varying assessment methods [27].

### Overview of disability support services and recipients

Some recent data [28] about the program reveal that:

• almost one quarter of a million (245,746) people used government funded disability support services in 2007–08. Disability support services are used predominantly by people under age 65 because of the target group of the time;

• close to 11,000 outlets delivered services in 2007–08, predominantly non-government organizations receiving government funding;

• government expenditure on disability support services during 2007–08 was $4.8 billion;

• the DSP was the main source of income for most service users aged 16 years and over since 2003-04 (66–75%).

**Figure 2:**
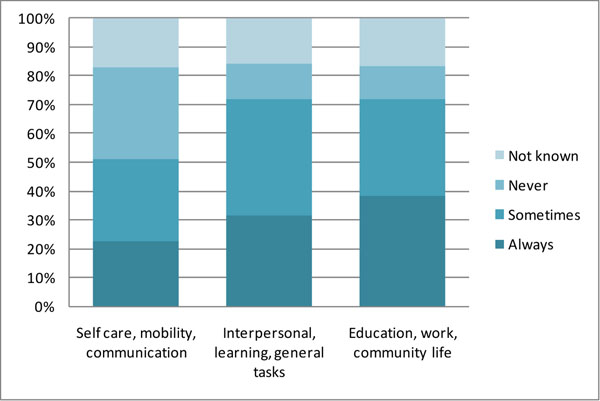
**Frequency of need for support in Activities & Participation domains, 2007-08** The figure shows the proportions of disability support service recipients who needed different frequencies of support in nine life areas based on the ICF Activities and Participation domains; the nine life areas are grouped into three groups for ease of display. The 2007-08 figures are Australia-wide, for 245,746 people receiving government funded disability support services. Source: [28] page 40.

The support needs of recipients were relatively high (Figure 2):

• almost 70% of service users needed support in education, work and/or community life;

• around 70% needed support in interpersonal interactions and relationships; learning, applying knowledge and general tasks and demands; and domestic life;

• some 50% needed support in self care, mobility and/or communication.

This compares with 6.3% of people of all ages in the general population who needed assistance with self care, mobility and/or communication in 2003 [4]. Of service recipients needing support, almost half needed support ‘always’ in order to carry out the activity or to participate in that area of life, or else were unable to do so at all.

### Eligibility for disability support services and the ICF

Supporting people in all areas of life relates to the broad goals of the National Disability Agreement and those of the UN Convention, notably Article 19 asserting the right to live independently and be included in the community**.** Since the new National Disability Agreement no longer describes the target group in terms of just three ICF domains, the way is open to using the full spectrum of activities and participation in devising any new assessment and eligibility criteria; this is imperative in the light of Australia’s ratification of the Convention. There are, broadly, two options. The first option would be to develop new, specific assessment tools that relate to the ICF as a framework and to the existing data capture framework for disability support services (see Figure 1). Alternatively, the many agencies involved in providing services could be enabled to continue using existing tools, but with a program of work to evaluate these so as to relate them more specifically to the ICF framework and the existing data capture framework; this could ultimately result in rationalization, for instance creation of a list of relevant assessment tools.

## ICF implementation in Australian data collections

As ICF is the international standard classification for functioning and disability, the two main national statistical organisations – the Australian Bureau of Statistics and the Australian Institute of Health and Welfare – use it in national data collections. An Australian User Guide was published soon after the ICF publication, with the aim of introducing the ICF and its potential, and encouraging its use [2].

Australian disability survey questions, a related census question, and related disability modules in health and social surveys are based on the ICF as an international standard (e.g. [4]). This consistency of disability concepts across social surveys and the census means that people with disabilities are ‘visible’ in the population, with the possibility of establishing information about their health, housing and economic status in comparison to the rest of the population. By using consistent disability concepts across disability and Aboriginal and Torres Strait Islander social surveys, it was possible to estimate disability rates among this numerically small but nationally important population group; adult Indigenous people were found to have more than twice the rate of disability of other Australians [29].

Australia’s system for setting national data standards in the health and community services sectors was established to promote consistency in these sectors [30]. ICF-related national data standards are available on line, comprising a suite of metadata items covering all dimensions and domains of the ICF, and including its qualifiers [26]. The benefits in using such standards are numerous – notably efficiency in design effort and the possibility of building a coherent statistical system that provides information about functioning in whatever setting, with each source adding to integrated national knowledge. The value of ensuring that population data (indicating need and demand) and disability services data (on supply) are based on the same concepts has been illustrated by studies of unmet demand for disability support services which have highlighted the need for new funding (e.g. [31,32]).

## Discussion

There is a great need for an integrated approach to disability policy and information, reflecting the three components of the Italian project [5]:

• legislation and a high level philosophical framework and policy guide;

• a technical framework that can underpin specific policies and programs aiming to achieve the major goals; and ,

• a language and set of tools, relating to both the above, that provides infrastructure for assessment methods and information systems.

This discussion comments on these three components, referring to Australian experience and future challenges.

### A high level philosophical framework and policy guide

The UN Convention, in Australia, acts as a philosophical and moral statement and framework guiding integrated and strategic policy; as yet there is no use in specific legislation. Broad agreements and strategies relating to the Convention are in place – the National Disability Agreement (among the national and state governments) and the National Disability Strategy, described previously. The Australian Human Rights Commission (with responsibilities for the Disability Discrimination Act) also has significant responsibilities in relation the Convention [33,34]. It is not only governments who are taking coordinated action. Disability organisations are partnering to compile a Shadow Report on Australia’s implementation of the Convention, with the aim of making recommendations directly to the United Nations Committee on the Convention [35].

The relationships among the three major components listed above are being explored in the Australian context (e.g. [36]). For many Articles of the Convention, significant corresponding concepts and items can be found in the ICF components of Activities and Participation and Environmental Factors. Connecting further, from the Articles of the Convention and the ICF to the Australian services system, it can be shown that the ICF provides links between the broad goals of the Convention and the details of the service system – both generic and specialist services.

The broad, inclusive and enabling approach to disability, in both the Convention and the ICF, are essential ingredients for definitions. Article 1 of the Convention refers to ‘physical, mental, intellectual or sensory impairments’ and provides perhaps a general statement of inclusion rather than a precise definition of disability; there will at times be a need for a more technical approach most appropriately using the ICF. It may be, moreover, that for a range of policy and advocacy reasons, some impairment-oriented language is desired; for instance, the guidelines on monitoring the UN Convention (released in late 2009) require that the treaty-specific report should contain impairment-based disability groupings: ‘Statistical data on the realization of each Convention right, disaggregated by sex, age, type of disability (physical, sensory, intellectual and mental), ethnic origin, urban/rural population and other relevant categories, on an annual comparative basis over the past four years’ [37]. Similarly, in Australia, in developing the Disability Services National Minimum Data Set (outlined previously), it was agreed by stakeholders that an item titled ‘disability group’ should be included (for instance, intellectual disability, physical disability, etc). This item is defined thus [38,26]:

***‘The disability groupings *** *are a broad description of similar experiences of disability and patterns of impairments, activity limitations, participation restrictions, support needs and related health conditions. ‘Disability group’ is not a diagnostic grouping, and there is not a one-to-one correspondence between a health condition and a disability group’.*

### A common technical framework for the evaluation of disability

Disability affects many people and, according to the ICF, human functioning and disability can be experienced, described and ‘measured’ on a continuum. There is no universal dichotomy splitting the population into ‘disabled’ and ‘not disabled’ and, by definition both in the Convention and the ICF, disability varies with environment or context. ‘Definitions’ within particular policy settings are, then, locations or thresholds on this spectrum rather than definitions of disability itself. So, unless we are satisfied with an unconnected set of policies, programs and related information, a common technical framework is essential.

Eligibility and assessment in Australia relate to any or all of ICF components, as outlined in this paper. In the income support system, eligibility measurement is evolving. The older ‘impairment’ evaluation is now operating alongside the newer JCA, with its functional focus. ‘Impairment’ however remains a mixed concept – of diagnosis and some aspects of functioning. Better information could be produced from the income support system by ‘deconstructing’ existing records to produce ICF-based information about:

• medical conditions, coded using the ICD and tabulated in ways comparable to other national data such as health surveys or hospital statistics;

• ‘work capacity’ or ‘work difficulty’ (the JCA), related to domains of the ICF, e.g. to determine the competencies for (re)employment as well as the problems which establish the entitlement to the DSP;

• environmental factors that act as barriers and should be remediate to assist people to participate in work.

Records organised in this way would provide greater clarity and useful information that could be related to other sources. For instance, the recipient profile could be related to other information about people with health conditions and functioning difficulties, enabling greater understanding of the recipient profile in the broader Australian context and over time.

The disability support services system, in contrast, has followed the international and national data standards. It is thus possible to relate to the ICF and ICF-related national data standards: the service target group specified in Agreements to 2007, key data items in the related data collection on services, and national disability surveys. The reward for this effort is that integrated policy analysis is enabled, for instance the studies of unmet need for services previously outlined. Any new national assessment criteria, for support services or long term care, should use the full spectrum of activities and participation (not just self care, mobility and communication); this is imperative in the light of Australia’s ratification of the Convention as well as the stated goals of the National Disability Agreement.

Criteria for and decisions about eligibility for long term benefits or care rely on judgment and prediction. A fair system requires relevant criteria, fit-for-purpose measures, and evidence-based application. It is essential that disability measures be related directly to the policy purpose and be well-tested for this relationship:

• As outlined in this paper, the relationships between the health condition (psychiatric disorders) and working, and environmental factors and working is not well evidenced, making the judgment about future working ability particularly difficult. More generally, the question arises as to the extent to which the Impairment Tables (Schedule 1B) are evidence-based, in terms of their ability to predict future work capacity.

• The cost of developing disability evaluation tools can be considerable, but applying ‘ready-made’ tools to the wrong measurement question can also be costly. A recent example of this risk is in a report proposing that a new long term care scheme focus on ‘severe or profound disability’ – an idea based on a survey construct meaning needing help in self care, mobility and/or communication [39] – without consideration of the effect that the use of this construct would have on current clients who have high support needs across all ICF domains of Activities and Participation. There are general risks in transferring measurement concepts from one field to another, for instance, transferring adult frameworks to apply to children; a recent study of children’s well-being in the UK acknowledged the need for further effort to understand the perspectives of children, for instance in constructing measures of well-being [40].

The eligibility concepts of the two major Australian disability programs described in this paper can, then, be related to any or all of the ICF components, but there is scope for improved clarity and better information. This information would, over time, build a stronger evidence base about the relationships among health conditions, participation restrictions (including work-related functioning difficulties) and predictions about future participation.

### ICF as a language supporting a disability evaluation framework, and a frame for the electronic health and social record of people with disability

The Australian experience with disability support services illustrates the ability of the ICF to underpin an integrated approach relating policy parameters, disability data and individual records. The recording of ‘support needs’ in the national data collection is achieved via a data capture framework useable across thousands of disability support services across the country. A ‘statistical linkage key’ enables records to be linked from year to year creating a large national database of individualised yet anonymised records about people’s disability and their access to disability services [28,38].

The use by the Australian Bureau of Statistics of a ‘disability module’ in health and other social surveys, related to the ICF and the main disability survey, and the 2006 census question on disability, enables a social record for the nation to be assembled covering a wide variety of topics.

It would be of significant national value in Australia, especially as a ‘continuum of care’ is desired, if these efforts toward consistency of concepts and information were expanded. It would thus be possible to obtain consistent data from health, aged care, disability and community services systems about key aspects of health and functioning.

Future directions and challenges include:

• The involvement in design of information systems of people with disabilities and service users is essential: information is then more robust, relevant and person-centred if service users are involved adequately in the design of information systems. This is not a new idea, has been advocated by people with disabilities for many years, and is increasingly recognised in the research field. For instance, activity and participation oriented definitions of disability have been found more culturally appropriate in indigenous communities in Africa where what one does to contribute to the good of the collective (e.g., extended family, village, community ) is highly valued, and more than the functional-medical-technical aspects of a disability [41];

• Including environmental interventions in more services and operationalising the ICF Environmental Factors components in all data collections (both administrative collections and surveys) is a challenge in Australia as in many other countries. The research community could become more active in suggesting practical ways of doing this.

• Building on the existing national information infrastructure is essential to help operationalise the broad intent of the National Disability Agreement and to monitor outcomes from the Convention. The requirement to monitor service accessibility for people with disability can be met by the inclusion of disability indicators in generic service information systems, for which the ICF and the related Australian data standards should be used.

• Many countries are now assembling indicator sets to monitor the well-being of the population, the outcomes from the service system, and progress in implementing the UN Convention. Traditionally, many indicator sets can be overly focussed on short term policy, ‘bunched’ around available data, and can miss important outcomes. Indicator sets that deal with broad questions of rights and well-being must be based on clear philosophical and conceptual frameworks such as those provided by UN Conventions [42].

## Conclusions

Overall, the UN Convention provides the philosophical vision and the call to action; the ICF provides a neutral framework for functioning and disability, consistent with these ideas. Being constructed as a classification, the ICF also provides infrastructure for measurement and assessment, and for relevant statistics to be designed and gathered, to monitor individual programs and a country’s overall implementation of the Convention; it is a tool that requires constructive, active and creative use. Without attention to all three components of the Italian project [5] and continuing effort to meet the challenges identified in this paper, it will not be possible to determine whether the goals of Australia’s National Disability Agreement or the ambitions of the Convention are being achieved.

## Competing interests

The authors declare that they have no competing interests.

Disclaimer: The views expressed in this paper are those of the authors and not necessarily those of the University of Sydney.
